# Embryonic thermal manipulation has short and long-term effects on the development and the physiology of the Japanese quail

**DOI:** 10.1371/journal.pone.0227700

**Published:** 2020-01-23

**Authors:** Anaïs Vitorino Carvalho, Christelle Hennequet-Antier, Sabine Crochet, Thierry Bordeau, Nathalie Couroussé, Estelle Cailleau-Audouin, Pascal Chartrin, Veerle M. Darras, Tatiana Zerjal, Anne Collin, Vincent Coustham

**Affiliations:** 1 BOA, INRAE, Université de Tours, Nouzilly, France; 2 Laboratory of Comparative Endocrinology, Department of Biology, KU Leuven, Leuven, Belgium; 3 GABI, INRAE, AgroParisTech, Université Paris-Saclay, Jouy-en-Josas, France; Tokat Gaziosmanpasa University, TURKEY

## Abstract

In vertebrates, the embryonic environment is known to affect the development and the health of individuals. In broiler chickens, the thermal-manipulation (TM) of eggs during the incubation period was shown to improve heat tolerance at slaughter age (35 days of age) in association with several modifications at the molecular, metabolic and physiological levels. However, little is known about the Japanese quail (*Coturnix japonica*), a closely related avian species widely used as a laboratory animal model and farmed for its meat and eggs. Here we developed and characterized a TM procedure (39.5°C and 65% relative humidity, 12 h/d, from days 0 to 13 of incubation) in quails by analyzing its short and long-term effects on zootechnical, physiological and metabolic parameters. Heat-tolerance was tested by a heat challenge (36°C for 7h) at 35 days of age. TM significantly reduced the hatching rate of the animals and increased mortality during the first four weeks of life. At hatching, TM animals were heavier than controls, but lighter at 25 days of age for both sexes. Thirty-five days after hatching, TM decreased the surface temperature of the shank in females, suggesting a modulation of the blood flow to maintain the internal temperature. TM also increased blood partial pressure and oxygen saturation percentage at 35 days of age in females, suggesting a long-term modulation of the respiration physiology. Quails physiologically responded to the heat challenge, with a modification of several hematologic and metabolic parameters, including an increase in plasma corticosterone concentration. Several physiological parameters such as beak surface temperature and blood sodium concentration revealed that TM birds responded differently to the heat challenge compared to controls. Altogether, this first comprehensive characterization of TM in Japanese quail showed durable effects that may affect the response of TM quails to heat.

## Introduction

In vertebrates, the embryonic environment affects the development and health of individuals from birth to adulthood [1]. Indeed, the embryonic environment can be influenced by a wide range of stressors including nutrition, disease, endocrine disruption, maternal stress [1] and temperature [2].

As birds are oviparous, the incubation conditions can be modified in order to study the impact of environmental challenges or stressors during embryogenesis, thus reducing the impact of the maternal influence. Incubation parameters such as temperature [2], light [3] or noise [4] are known to impact development and behavior. In a context of climate change, with an increase of global average temperature and the occurrence of extreme heatwaves [5], numerous studies have reported the utilization of the thermal-manipulation (TM) of eggs during the incubation period to improve the later-life heat tolerance of chickens [2,6–16] and turkeys [17,18] or to modify muscle development in ducks [19–21]. The TM consists in a modification of the incubation temperature of avian eggs during embryogenesis, which is often cyclic and applied during defined periods of the embryonic development. For instance, one protocol of TM used in chicken involved a cyclic increase of 1.7°C of the incubation temperature (12 h/day) from day 7 to day 16 of the incubation period [9]. The TM protocols were shown to have a long-term impact on the performance (weight gain, muscle yield, etc.), the physiology (thyroid axis function, acid-base balance, respiratory process, etc.), the metabolism (glucose metabolism, regulation of mitochondrial function, etc.), the regulation of gene expression and the epigenome landscape [7,9,10,12–14,22,23].

In previous experiments performed on chickens, control (C) and TM birds were subjected to an acute heat challenge (HC) at five weeks of age to investigate their response to a heat stress [9,22]. The HC protocol consisted in increasing the rearing temperature (from 21°C to 32–35°C), for a short period (5h) [9,22]. The HC revealed that TM chickens were more tolerant to heat by reducing mortality during a heat challenge by 50% [22]. This tolerance was associated with physiological, metabolic and cellular changes, such as the regulation of surface temperature of peripheral body parts involved in the body thermoregulation and the heat dissipation [24,25], a lower plasma corticosterone concentration in TM birds (a hormone associated to stress response in birds [26]) and modifications of the expression of genes whose function is associated to metabolism, stress response, vascularization, anti-apoptotic and epigenetic processes [2,9,22].

The Japanese quail (*Coturnix japonica*) is an agronomic species of interest closely related to chickens [27,28] and a primary avian model in developmental biology [29] as well as in genetic and genomic analyses [30]. Furthermore, while some effects of short TM-like treatments on quail physiology and metabolism have been previously reported, to date there is no comprehensive study of the impact of TM for this species [31–34]. Thus, in order to gain new insights about the physiological and metabolic effects of TM in birds, we characterized a model of TM on a highly inbred line of Japanese quails.

Based on the previously described protocol in chickens [22], we applied a TM treatment consisting of a 1.7°C increase for 12 hours per day from the first day of incubation to day 13 of incubation (I0-13). Treatment was applied earlier during the embryonic development as the early stages of this development are known to be highly susceptible to environmental parameters [1,35]. The short and long-term effects of TM on zootechnical, physiological and metabolic parameters known to be impacted in the other avian models, such as mortality, weight, surface and internal temperatures, as well as hematological parameters [7,9,10,12–14,22] were analyzed from the incubation period to the onset of sexual maturity (35 days of age–D35). Furthermore, in order to investigate the heat stress response of TM quails, a heat challenge (HC) protocol, corresponding to a moderate heat stress [36,37], was performed at D35 by an increase of the environmental temperature to 36°C for 7 hours. Whereas previous analysis of TM in chickens principally focused on the male response [9,13], here we investigated the reaction of the both sexes to the TM treatment or/and to the HC protocol. Collectively, our data revealed that TM had short and long-term impacts on the physiology and metabolism of quails and that TM animals responded differently to heat stress compared to C quails.

## Materials and methods

### Experimental design and data collection

All experiments were carried out in accordance with the legislation governing the ethical treatment of birds and were approved by the French Ministry of Higher Education and the Val-de-Loire Animal Ethics Committee (authorization N° APAFIS #4606–2016032111363124).

Animal rearing was performed in the PEAT INRAE Poultry Experimental Facility (2018, https://doi.org/10.15454/1.5572326250887292E12).

**Fig 1:**
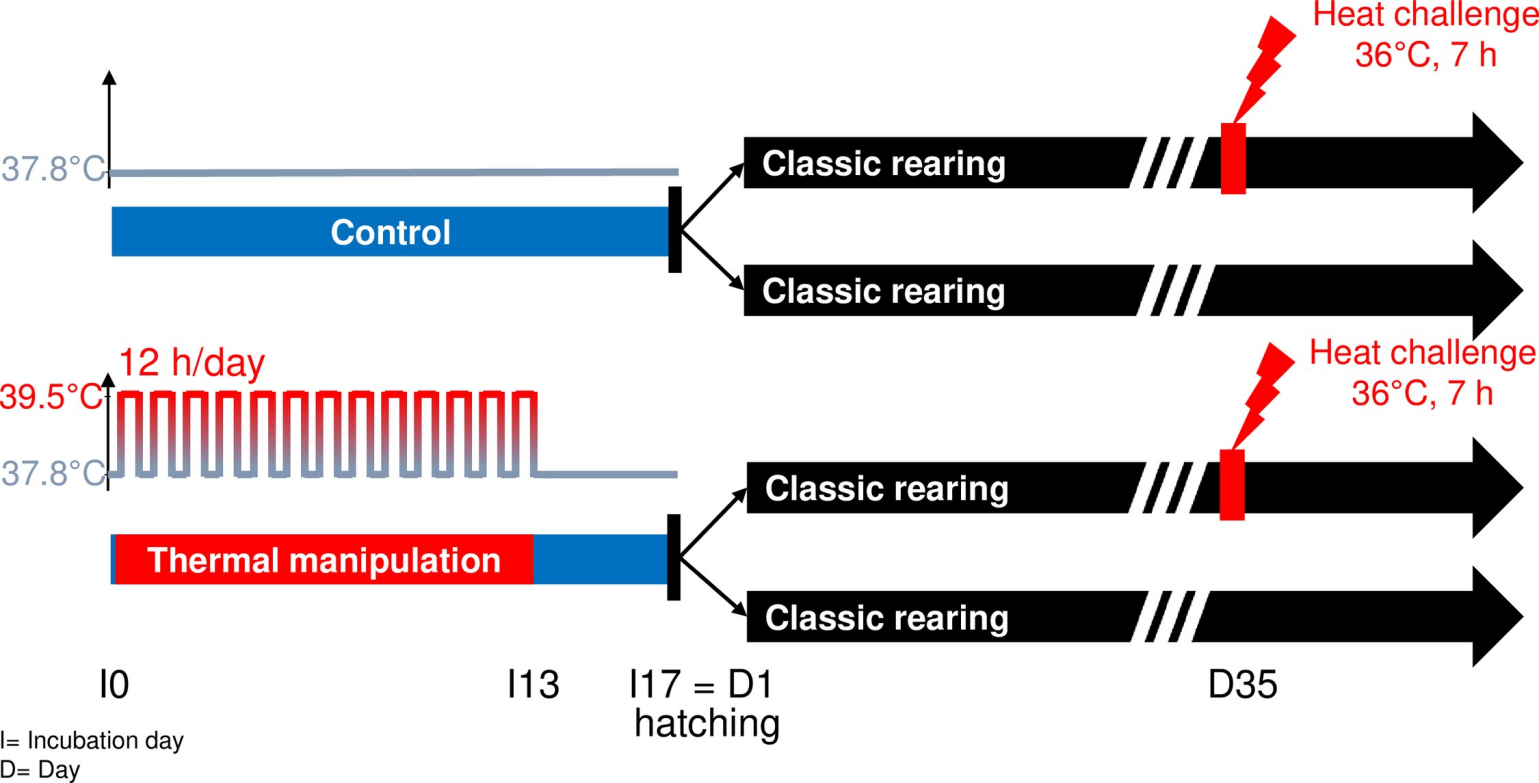
Experimental protocol of thermal manipulation in quail. Cons DD quail eggs were incubated in control (C) conditions, *i*.*e*. maintained at 37.8°C and 56% relative humidity (RH) during the whole incubation period (upper graph), or thermo-manipulated (TM) with a cyclic increase of incubation temperature at 39.5°C and 65% RH for 12 h/d from incubation days I0 to I13 (lower graph). Hatching corresponds to both I17 and D1. Red boxes under arrows indicate the heat challenge performed at D35 (36°C for 7h).

In this study, we used the Cons DD Japanese quail line, a highly consanguineous line established at INRAE [38]. Eggs were collected from 80 Cons DD quails during 10 days and stored at 16°C before incubation. Eggs produced by each couple were dispatched equally between treatments to obtain homogeneous egg groups: 402 eggs with an average weight of 10.25 g ± 0.83 for the C incubation group and 414 eggs with an average weight of 10.27 g ± 0.82 for the TM incubation group. Eggs were then incubated in two identical automated commercial incubators with regulation of temperature, humidity and ventilation (Bekoto B64-S, Pont-Saint-Martin, France). Incubation parameters were recorded during the whole incubation period and temperature was confirmed by two independent KIMO KT-220 probe thermometers (KIMO, Montpon Ménestérol, France). Control eggs (C) were maintained at 37.8°C and 56% relative humidity (RH) during the whole incubation period in the first incubator. TM was applied by increasing the incubation temperature to 39.5°C and 65% RH for 12 h/d from I0 (from the 12^th^ hour of incubation—around the developmental stage 3 of the Hamburger & Hamilton scale corresponding to the intermediate primitive streak formation) [39–41] to I13 (Fig 1), in the second incubator. The incubation of C eggs began 12 h before the incubation of TM eggs to synchronize hatching, 12 h corresponding to the acceleration of embryonic development induced by heat [22,42]. All eggs were subjected to a rotation of 90 degrees every hour. The presence of living embryos was checked at I5 and I11 by candling. At I14, all eggs were transferred to one hatcher and incubated at 37.5°C with RH between 75 and 80% until I18. Hatched quails without body defects were collected, weighed and identified with a numbered wing tag at I17 (the peak of the hatching rate, also considered as the start of out-shell life, D1–262 C and 243 TM). Zootechnical parameters, such as the proportion of dead-in-shell embryos (eggs with no trace of pipping, reflecting mortality between I11 and I18) or embryos with external pipping (eggs incompletely hatched), were evaluated at I18 (S1 Table).

The timing of hatching may be influenced by the incubation temperature [22,42]. Therefore, only the quails (without body defects) that hatched between I16 4 pm and I17 12 pm were transferred to a single concrete-floored room covered with litter and reared in standard conditions: the temperature was gradually decreased from 40°C at the first day of rearing (D1) to 20°C at D24 and water and food were supplied *ad libitum*. The mortality was recorded every day and the early life mortality was calculated at D28 on quails still wearing their identification tag (256 C and 223 TM, S2 Table). At D25, all animals were sexed visually.

For the further analyses, only quails hatched at I17 with a non-ambiguous phenotypic sex at D28 and a wing identification tag at D35 were included in the statistical analysis.

At D11 and D21, internal temperatures were measured using a KIMO KTT-310 probe thermometer inserted into the cloaca of a randomly selected set of animals (63 C and 56 TM at D11; 67 C and 68 TM at D21) (S1 File). At D25, quails were weighed. At D35, an HC procedure was applied on a randomly selected subset of C and TM quails with an approximately equal distribution of sexes by transferring them to a room heated at 36°C for 7 hours (71 C-HC and 57 TM-HC; Fig 1). This temperature had previously been shown to induce a moderate heat stress response with hyperthermic responses (including panting) in Japanese quails [36,37]. The remaining non-challenged quails were transferred into another room and kept under standard conditions at 20°C (81 C-RT and 79 TM-RT) until measurement and sampling. During the HC, water and food were supplied *ad libitum* and quail behavior (panting, food and water intake, or prostration) was visually monitored by a video camera. Sampling was performed in the morning for RT birds while HC birds were subjected to the HC. HC quails were then sampled in the afternoon of the same day. This difference in sampling time between RT and HC quails is supposed to have a limited impact on the physiological (including the temperature) and metabolic analyses, since previous studies revealed that these parameters remain stable during the day in quails [43–45] and that daytime has no clear effect on the plasma corticosterone concentration [44] and blood T4 hormone levels in chickens [45,46]. The internal and body surface temperatures were measured on randomly selected animals (74 C-RT, 73 TM-RT, 66 C-HC and 54 TM-HC). The internal temperature was measured as described for D11/21 and the body surface temperature was measured by thermal imaging using an FLIR B335 infrared digital camera (Wilsonville, USA). Both measurements were performed within 5 minutes after removal from the rooms containing the birds. The body surface temperature was collected for several body parts reflecting the internal body temperature, such as the eye, or implicated in heat dissipation in birds, i.e. the ear, beak and shank (S1 Fig) [24,25]. The weight was evaluated directly after HC at D35 on the same subset of quails. For the hematological analysis, blood samples were collected once on a subset of randomly selected quails (37 C-RT, 33 TM-RT, 34 C-HC and 30 TM-HC).

### Thermal imaging analysis

From the pictures obtained using the infrared camera, the surface temperatures of three parts of quail bodies (eye, ear and shank) were measured using the FLIR Tools software (version 5.13.17214.2001, FLIR Systems). The ear and eye surface temperatures were evaluated respectively as the hottest and the coolest points of the head (S1 Fig). The surface temperature of the shank was evaluated as the mean temperature of a line drawn in the middle of the shank (S1 Fig). The surface temperature of beaks was evaluated as the mean surface temperatures were measured using the ThermaCAM Researcher Pro 2.10 software as previously described [47] (S1 Fig).

## Hematological analyses

### Blood gases, electrolytes and hormones

One mL of blood was sampled from a subset of randomly selected quails (18 C-RT, 17 TM-RT, 16 C-HC and 14 TM-HC) using a heparinized needle and syringe (SS02SE1, Terumo, Guyancourt, France; BD Microlance^™^ 3 23G, BD, Le Pont-De-Claix, France) for blood gas, electrolyte and hormone measurements. Blood gases and electrolytes were evaluated from 0.5 mL of blood using an IRMA True Point blood analysis system at room temperature single-use CC cartridges (ITC Nexus DI, Edison, NJ). Partial pressure of carbon dioxide (pCO2) and oxygen (pO_2_), pH, hematocrit (Hct), sodium (Na^+^), potassium (K^+^), ion calcium (iCa), bicarbonate (HCO_3_^-^), total carbon dioxide (TCO_2_) concentrations, base excess in blood (Beb), base excess in extra-cellular fluid (Beecf), oxygen saturation percentage (O_2_sat), and total hemoglobin (tHb) were measured. The remaining 0.5 mL of blood, transferred into a heparinized collection tube, was used to measure plasma concentrations of thyroid hormones and corticosterone. Plasma was separated by centrifugation at 3 000 × *g* for 10 min at 4°C and plasma triiodothyronine (T3) and thyroxine (T4) concentrations were measured by RIA as previously described [48]. The antisera for T3 and T4 were purchased from Byk-Belga (Brussel, Belgium). Plasma corticosterone concentration was measured using a commercial double antibody RIA-kit (n° 07–120103, MP Biomedicals, NY, USA).

### Blood glutathione and derivative levels

One mL of blood was collected using heparinized needle, syringe and collection tube from another subset of quails (19 C-RT, 16 TM-RT, 18 C-HC and 16 TM-HC). Plasma was separated from the erythrocyte pellet by centrifugation at 3000 × *g* for 10 min at 4°C. Fifty μL of the erythrocyte pellet were used to evaluate glutathione and derivative levels, glutathione peroxidase activity and total antioxidant status. Levels of glutathione (GSH) and its oxidized derivate (disulfide dimer, GSSG) were evaluated on erythrocyte lysate using a Glutathione Assay Kit according to the manufacturer’s protocol (ref. 703002, Cayman Cheminal Compagny, Ann Arbor, Michigan, USA). Plasma was divided into two fractions: one for the measurement of metabolites (uric acid, triglycerides, and glucose, 2 x 100 μL) and the other for the measurement of thiobarbituric acid reactive substances (TBARS, 250 μL). Total antioxidant status and glutathione peroxidase activity were evaluated using a total antioxidant assay kit and RANSEL assay respectively, according to manufacturer’s protocol (respectively NX 2332 and RS 504, Randox, Roissy-en-France, France). Plasma uric acid, triglyceride and glucose concentrations were measured using commercial colorimetric kits (respectively, MG981788, MG981786, MG981780, Thermo Fisher Diagnostics S.A.S, Asnières-sur-Seine, France). TBARS measurement was performed as previously described [49].

### Statistical analyses

All statistical analyses were performed with R software version 3.5.3 [50] using the following packages: nlme (version 3.1–137, [51]), lsmeans (version 2.30–0, [52]) and multcomp (version 1.4–10, [53]). Statistical testing was performed on datasets at a statistical significance of 5%.

### Fertility and mortality during incubation and during the first four weeks of life

During egg incubation, the impact of TM on fertility, embryonic mortality, external pipping, hatchability (calculated on the number of fertile eggs) and observed body defects was analyzed with a Chi2 test on 402 C and 414 TM eggs at I18 (one day after the hatching day, I17). The impact of TM on the total early life mortality (corresponding to the first four weeks of life) was analyzed with a Chi2 test at D28 on quails still wearing their identification tag (256 C and 223 TM, S2 Table), as previously described.

### Physiology data at D35

Only quails hatched at I17, with a determined phenotypic sex at D28 and with a wing identification tag at D35 were included in the statistical analysis of the physiological data, i.e. 25 to 38 animals per sex and per condition to analyze the weight and temperatures, and 4 to 10 quails per sex and per group concerning the blood parameters and the hormone and metabolite concentrations.

The impacts of the sex, TM and HC on weight, temperature and blood parameters (including the levels of metabolites and hormones) were analyzed at D35 with a linear model (model 1).

The following linear model (model 1) was used:

$$Y_{ijkl}=\alpha_{i}+\beta_{j}+\gamma_{k}+{\left(\alpha \beta \right)}_{ij}+{\left(\alpha \gamma \right)}_{ik}+{\left(\beta \gamma \right)}_{jk}+{\left(\alpha \beta \gamma \right)}_{ijk}+\varepsilon_{ijkl}$$

where Y_ijkl_ is the observed variable measured on the bird l, α_i_ is the fixed effect of sex (i = female and male), β_j_ is the fixed effect of embryonic treatment (j = C, TM), γ_k_ is the fixed effect of HC at D35 (k = RT, HC), (αβ)_ij_ is the interaction effect of sex and embryonic treatment, (αγ)_ik_ is the interaction effect of sex and HC, (βγ)_jk_ is the interaction effect of embryonic treatment and HC, (αβγ)_ijk_: interaction effect of sex, embryonic treatment and HC, ε_ijkl_ is the random residual term ~ N(0, σ^2^).

Post-hoc analyses from model 1 with Tukey’s multiple comparisons adjustment were performed depending on the significant terms.

### Body weight measurements from hatching until D35

Since the HC had no impact on the body weight (analyzed with the previously described linear model, model 1), all birds (including the ones exposed or unexposed to the HC) were considered in a mixed linear model (model 2). In addition to the previously described selection of considered quails, only the birds with weight information at all stages (i.e. I17/D1, D25 and D35) were analyzed to estimate their individual weight evolution, corresponding to 140 C (72 females and 68 males) and 127 TM (60 females and 67 males).

To analyze the body weight, the following mixed linear model [54,55] was performed for modelling heteroscedasticity and correlated errors (model 2):

$$Y_{ijklm}=\alpha_{i}+\beta_{j}+\gamma_{k}+{\left(\alpha \beta \right)}_{ij}+{\left(\beta \gamma \right)}_{jk}+{\left(\alpha \gamma \right)}_{ik}+U_{lm}+\varepsilon_{ijklm}$$

where Y_ijklm_ is the observed body weight of the bird l, α_i_ is the fixed effect of sex (i = female and male), β_j_ is the fixed effect of age (j = D1, D25 and D35), γ_k_ is the fixed effect of embryonic treatment (k = C, TM), their interactions are (αβ)_ij_, (βγ)_jk_, (αγ)_ik_ and (αβγ)_ijk_, U_lm_ ~ N(0, σ_U_^2^) is the random effect of the bird l within parents m and ε_ijklm_ is the random residual term ~ N(0, σ^2^|μ_ijklm_|^2δ^).

To highlight the impact of TM within a sex at a specific time, post-hoc analyses from the model 2 were performed to test the impact of embryonic treatment (TM versus C) with the factors sex and age set at fixed values (i.e. F-D1, F-D25, F-D35, M-D1, M-D25 and M-D35).

## Results

### Effects of TM on embryonic life and hatchability

No impact of the embryonic TM treatment was observed on the fertility rate and embryonic death at I5 and I11 (Table 1). TM reduced significantly the hatchability (Table 1). This decrease was associated with an increase of the rate of dead-in-shell quail chicks (Table 1). No impact of TM on body defects was observed (Table 1).

**Table 1:** Effects of thermal manipulation on zootechnical parameters during incubation and at hatching.

Embryonic treatment	C	TM	p-value
Fertile eggs	338	351	0.856
Embryonic death at I5	21	28	0.451
Embryonic death at I11	8	5	0.529
Total early embryonic death	29	33	0.807
External pipping	20	27	0.439
**Dead-in-shell**	20	38	**0.029**
**Hatched eggs at I18**	269	253	**0.027**
Quails with body defects	3	3	1

C: control (n = 402); TM: thermal manipulation during embryonic incubation (n = 414); I: incubation day. P-values lesser than 0.05 were considered as significant (in bold type). Individual data are presented in S1 Table.

### Impact of TM on quail mortality, growth and physiology from hatching to D35

**Fig 2:**
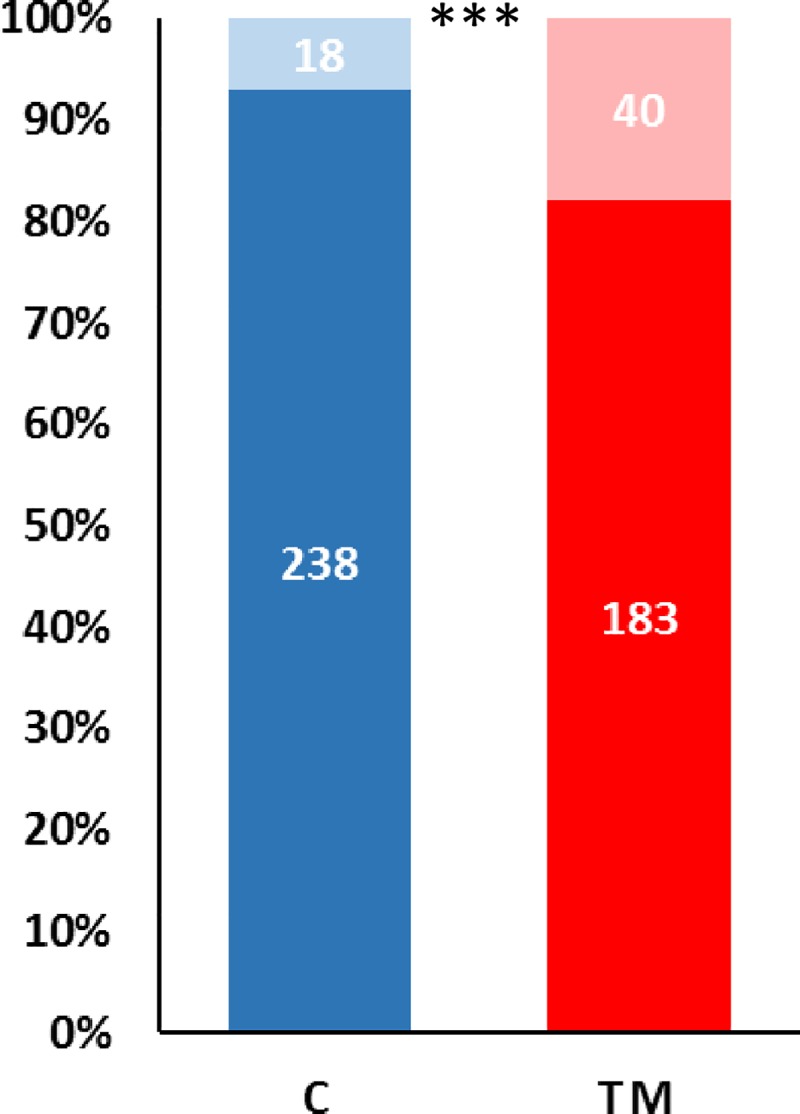
Effect of thermal manipulation on mortality during the first four weeks of age (from day 1 to day 28). C: control; TM: thermal manipulation during embryonic incubation. The bold parts of the bars represent the surviving quails and the light parts, the dead ones. ***: p-value < 0.001. Individual data are presented in S2 Table.

After hatching, quails were reared in standard conditions and mortality was measured between hatching and D28. We found that TM significantly increased quail mortality during this period (Fig 2).

**Fig 3:**
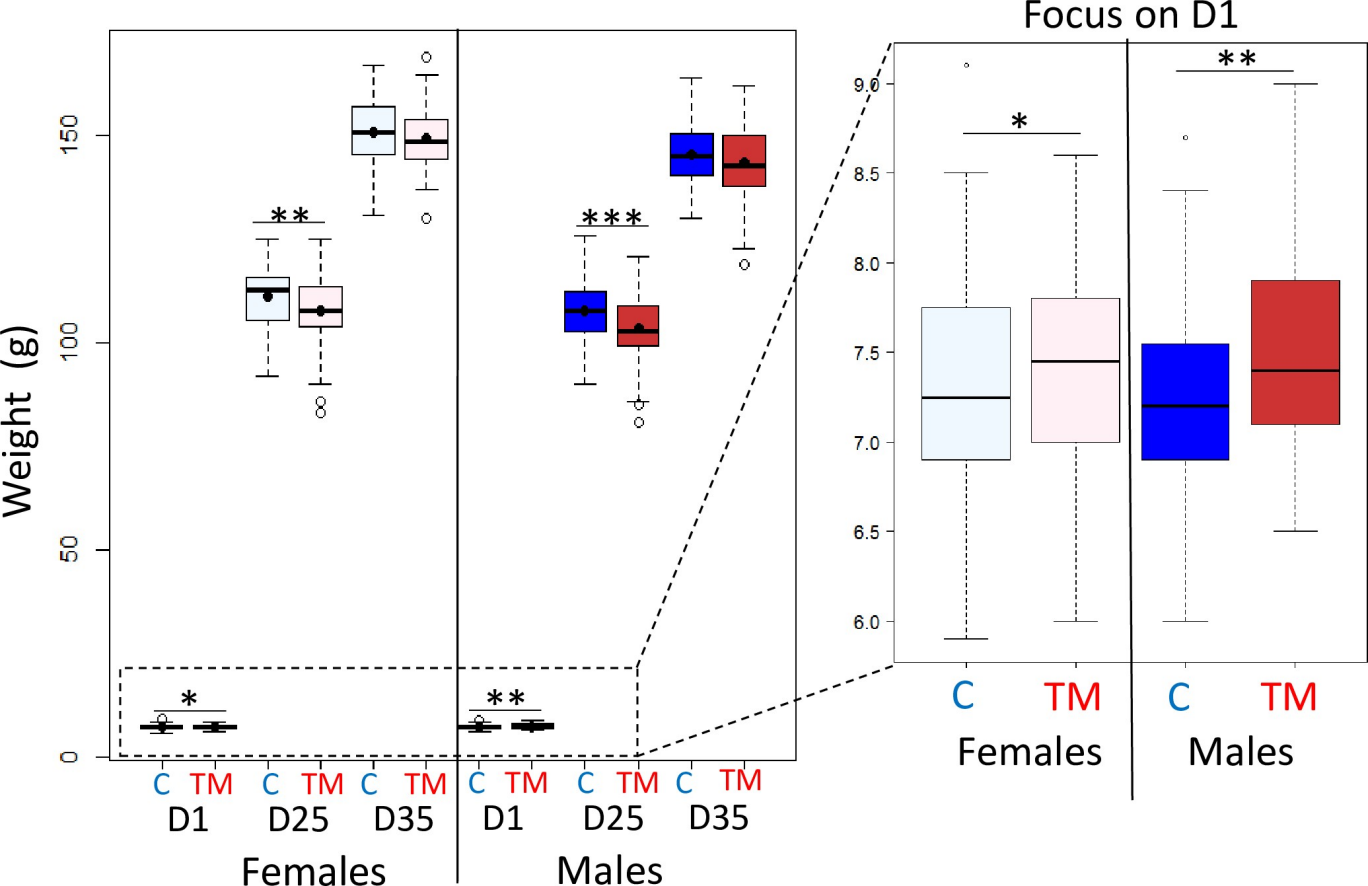
**Effect of thermal manipulation on quail weight (g) from day 1 (D1) to D35.** C: control; TM: thermal manipulation during egg incubation; D: day. Impact of the TM on quails of each sex and at D1, D25 or D35. The dots correspond to the mean. *: p-value ≤ 0.05, **: p-value ≤ 0.01, ***: p-value ≤ 0.001. Individual data are presented in S3 Table.

Quail growth was measured by weighing the animals at the day of hatching (I17/D1), at D25 and at D35. Between hatching and D35, TM interacted significantly with the age (Table 2). Post-hoc analyses revealed a significantly greater weight of TM animals observed for both female and male quails at hatching (Fig 3) compared to C, whereas at D25 TM quails were lighter than C birds (Fig 3). At D35, the impact of TM was no longer observed (Fig 3, Table 3). As expected, the interaction between sex and age was significant at 5% (p-value < 0.001) because of the growth of animals and the fact that, even if at hatching no weight difference was observed between females and males, females became heavier than males over time (Tables 2 and 3, Fig 3).

**Table 2:** Effects of sex, thermal manipulation and age on body weight from hatching to day 35.

	Statistics (p-value)						
	Sex	TM	age	Sex*TM	Sex*age	TM*age	Sex*TM*age
**Body weight**	0.387	**<0.001**	**<0.001**	0.642	**<0.001**	**<0.001**	0.771

P-values lesser than 0.05 were considered as significant (in bold). Individual data are presented in S3 Table.

**Table 3:** Effect of sex, thermal manipulation and heat challenge on quail weight, temperature, blood parameters and hormone and metabolite concentrations at day 35.

		Statistics (p-value)						
		Sex	TM	HC	Sex x TM	Sex x HC	TM x HC	Sex x TM x HC
Growth	**Weight (g)**	**<0.001**	0.057	0.271	0.919	0.494	0.955	0.772
Temperature	**Internal (°C)**	0.515	0.732	**<0.001**	0.210	0.992	0.665	0.984
	**Ear (°C)**	0.145	0.079	**<0.001**	0.382	0.961	0.127	0.700
	**Eye (°C)**	**<0.001**	0.202	**<0.001**	0.163	0.882	0.523	0.272
	**Beak (°C)**	0.471	0.746	**<0.001**	0.425	0.899	0.505	**0.001**
	**Shank (°C)**	0.491	**0.002**	**<0.001**	**0.041**	0.086	0.374	0.168
Blood parameters	pH	0.365	0.449	0.129	0.797	0.338	0.340	0.967
	pCO_2_ (mmHg)	0.637	0.773	0.330	0.544	0.716	0.211	0.782
	**pO**_**2**_ **(mmHg)**	0.229	0.102	**0.001**	**0.035**	0.193	0.757	0.573
	**O**_**2**_**sat (%)**	0.250	0.101	**0.002**	**0.022**	0.138	0.283	0.167
	**TCO**_**2**_ **(mM)**	0.518	0.593	**0.011**	0.884	0.506	0.866	0.732
	Hct (%)	0.577	0.145	0.257	0.362	0.784	0.591	0.470
	**Na+ (mM)**	0.172	0.252	**0.037**	0.841	0.661	0.427	**0.030**
	**K+ (mM)**	0.737	0.071	**0.011**	0.074	0.195	0.741	0.542
	**iCa (mM)**	0.532	0.169	**<0.001**	0.131	0.195	0.511	0.593
	**HCO**_**3**_**- (mM)**	0.503	0.590	**0.012**	0.896	0.486	0.888	0.724
	**Beb (mM)**	0.413	0.501	**0.016**	0.998	0.391	0.796	0.796
	**Beecf (mM)**	0.439	0.520	**0.014**	0.949	0.420	0.904	0.778
	tHb (g/dL)	0.538	0.149	0.247	0.390	0.760	0.579	0.408
	**Total antioxydant status (mmol/L)**	0.080	0.160	**0.001**	0.229	0.657	0.573	0.555
	Glutathione peroxidase (U/L)	0.461	0.379	0.277	0.233	0.748	0.973	0.180
	GSSG (μM)	0.228	0.822	0.498	0.391	0.897	0.567	0.197
	GSH/GSSG	0.572	0.517	0.698	0.715	0.871	0.497	0.894
	TBA (mmol/ml)	0.922	0.376	0.846	0.897	0.326	0.399	0.763
Hormones	**Corticosterone (ng/mL)**	0.673	0.951	**0.003**	0.659	0.206	0.739	0.236
	**T3 (pmol/mL)**	**0.039**	0.066	0.077	0.434	0.717	0.421	0.760
	**T4 (pmol/mL)**	0.512	0.517	**0.003**	0.838	0.826	0.347	0.839
Metabolites	**Uric acid (mg/L)**	0.719	0.469	**<0.001**	0.497	0.269	0.693	0.837
	**Glucose (mg/L)**	0.369	0.445	**0.010**	0.237	0.977	0.466	0.667
	Triglyceride (mg/L)	0.068	0.800	0.114	0.222	0.734	0.353	0.113

TM: thermal-manipulation treatment during egg incubation; HC: heat challenge. pCO_2_: partial pressure of carbon dioxide; pO_2_: partial pressure of oxygen; O_2_sat: oxygen saturation; TCO_2_: total carbon dioxide; Hct: hematocrit; Na^+^: ion sodium, K^+^: ion potassium; iCa: ion calcium; HCO_3_-: ion bicarbonate; Beb: base excess in blood; Beecf: base excess in extra-cellular fluid; tHb: total hemoglobin; GSSG: disulfide dimer of oxidized glutathione; GSH: reduced glutathione; TBA: thiobarbituric acid; T3: triiodothyronine; T4: thyroxine. P-values were presented and were considered as significant when lesser than 0.05 (in bold). Individual data are presented in S3 Table.

**Fig 4:**
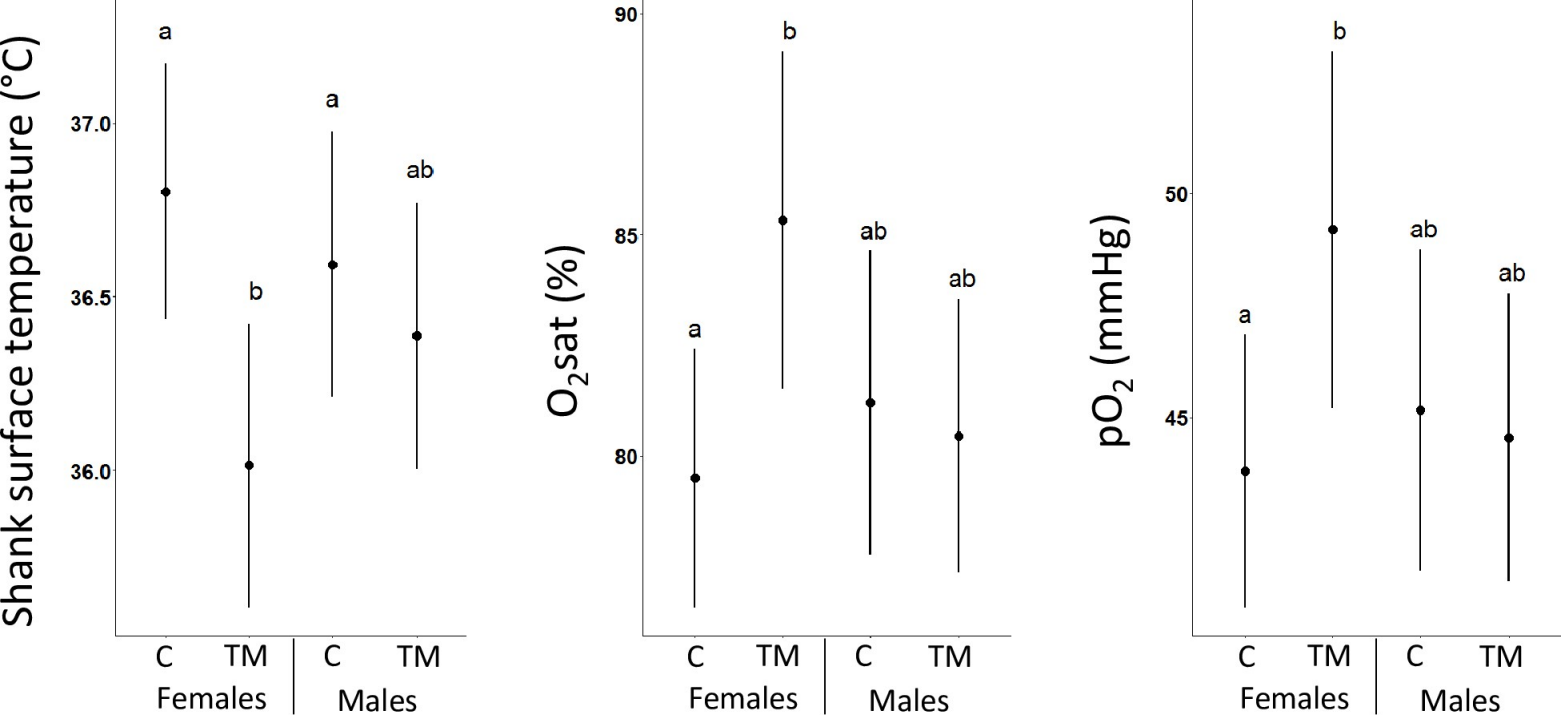
Effect of thermal manipulation and sex on shank surface temperature, oxygen saturation (O_2_sat) and partial pressure (pO_2_) at day 35. C: control; TM: thermal manipulation during egg incubation. The dots correspond to the mean and the range of the lines correspond to the confidence interval of the mean. Different letters indicate significant differences (p-value ≤ 0.05). Individual data are presented in S3 Table.

No statistically significant impacts of TM treatment, sex and their interaction were revealed for the internal temperature at D11 and D21 (S1 File) and D35 (Table 3). Furthermore, TM in interaction with sex had a significant impact on the shank surface temperature at D35 (Table 3), with a decrease of the shank temperature for TM birds compared to C birds for females only (Fig 4).

The hematological analysis also revealed a significant interaction between TM and sex for O_2_sat and pO_2_, with higher O_2_sat and pO_2_ in TM than in C in females only at D35 (Table 3, Fig 4).

### TM and HC impacts on quail temperature and physiology at 35 days of age

During the HC, no behavioral signs of distress (panting, reduction of food intake, increase of water intake and prostration) were observed.

**Fig 5:**
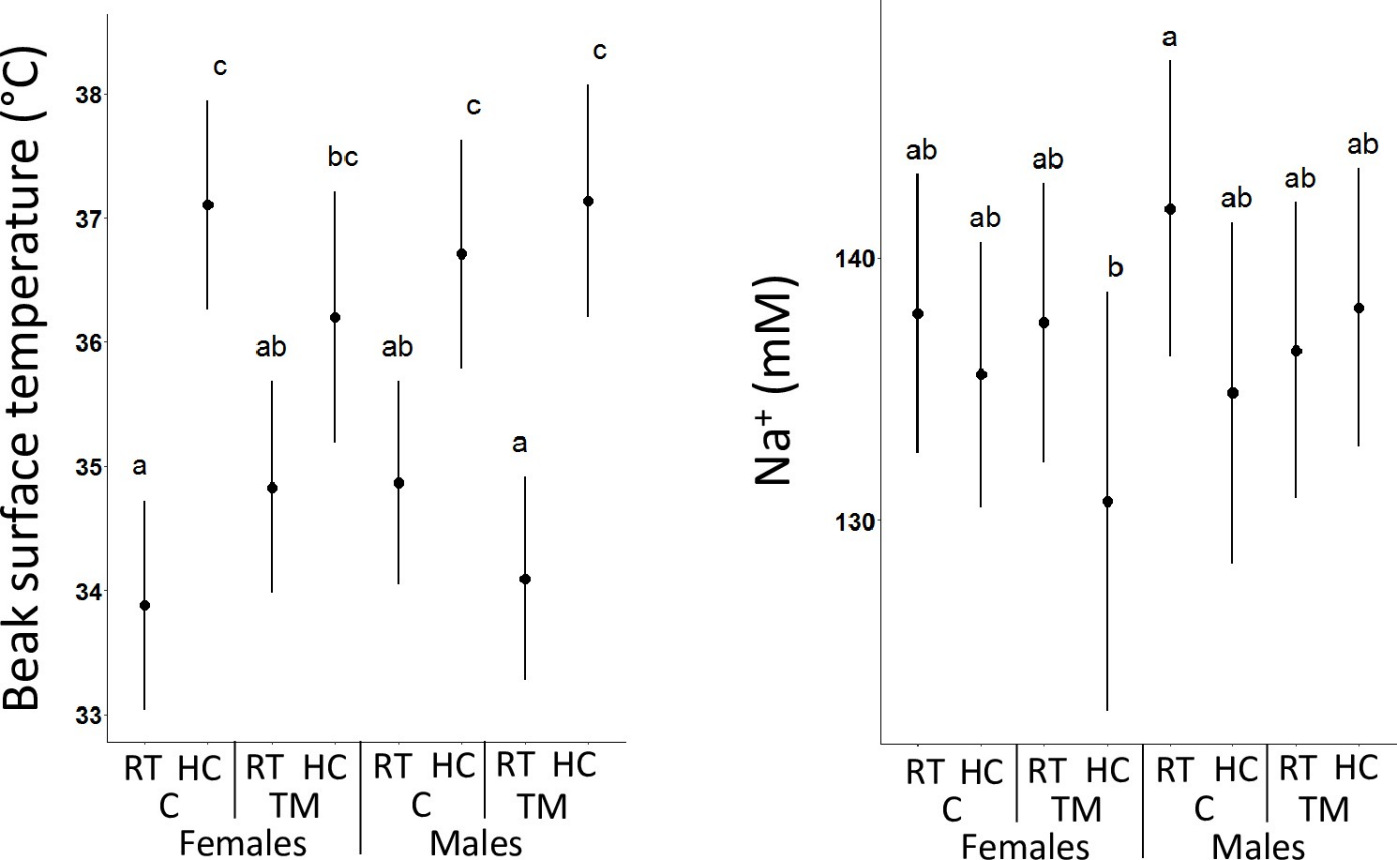
Effect of thermal manipulation, heat challenge and sex on beak surface temperature and plasma Na^+^ concentration at day 35. C: control; TM: thermal manipulation during egg incubation; RT: non-challenged birds; HC: heat-challenged birds. The dots correspond to the mean and the range of the lines correspond to the confidence interval of the mean. Different letters indicate significant differences (p-value ≤ 0.05). Individual data are presented in S3 Table.

**Fig 6:**
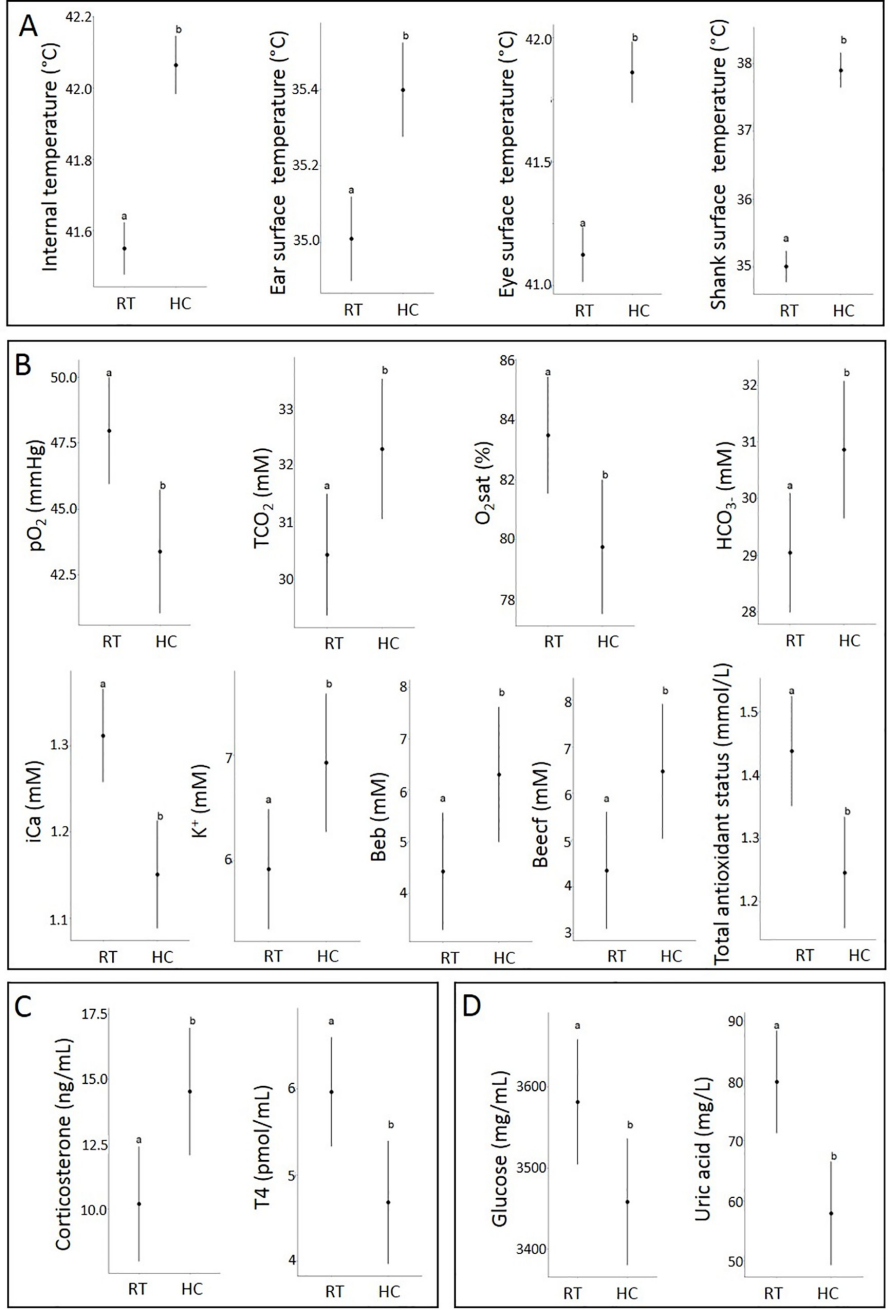
Effect of heat challenge on temperature (A), blood parameters (B) and hormone (C) and metabolite (D) concentrations at day 35. RT: non-challenged birds; HC: heat-challenged birds. TCO_2_: total carbon dioxide; K^+^: ion potassium; iCa: ion calcium; HCO_3_-: ion bicarbonate; Beb: base excess in blood; Beecf: base excess in extra-cellular fluid; T4: thyroxine. The dots correspond to the mean and the range of the lines correspond to the confidence interval of the mean. Different letters indicate significant differences (p-value ≤ 0.05). Individual data are presented in S3 Table.

At the end of the HC, the internal and surface temperatures of TM and C quails were measured (Table 3). The interaction between sex, TM and HC as well as HC as principal effects were significant for the beak temperature (Table 3), with an increase for all HC birds when compared to their counterparts under other conditions except for the TM females (Fig 5). As expected, all other temperature parameters (internal and surface) were significantly impacted by the HC (principal effect) with an increased temperature in HC animals when compared to their RT counterparts (Table 3, Fig 6).

Blood gas parameters, hormone and metabolite concentrations were estimated in blood samples of TM and C quails that were submitted to HC or not (Table 3, Figs 4, 5 and 6). TM had an impact in interaction with the HC and the sex on the concentration of Na^+^ (Table 3), with a higher sodium ion level in C-RT males compared to TM-HC females (Fig 5). Furthermore, HC, as principal effect, impacted several blood parameters as well as hormone and metabolite concentrations (Table 3). Thus, a significant increase of TCO_2_, HCO_3-_, K^+^, Beb, Beecf and corticosterone levels was observed in HC birds compared to C birds, whereas a decrease was observed for pO_2_ and O_2_sat as well as the concentration of iCa, total antioxidant status, T4, glucose and uric acid (Fig 6).

## Discussion

In this study, we characterized the short and long-term impacts of TM on Japanese quail physiology and development considering its important role as a laboratory model [29] and its economic impact in several countries [38]. We developed a TM procedure that consisted of a cyclic increase of egg incubation temperature (+1,7°C, 12 h/day) from incubation days I0 to I13.

No TM impact was observed on early embryonic mortality but the TM induced a significant decrease of hatchability. This may potentially be explained by an increase of embryonic mortality during the last third of incubation between I11 and hatching, reflected by the increase of dead-in-shell rate. A negative effect was not observed by Alkan and colleagues [31] but this difference can be explained by the shorter period of temperature increase in their study (either 3 days at the beginning or at the end of the incubation period). In chickens, TM treatments did not impact significantly hatchability except when the TM was applied 24 h/day, for which a severe decrease in hatchability was reported [13,42]. therefore we speculate that embryonic mortality may be linked to the early timing of cyclic increases at 39,5°C. Similarly, TM also increased the mortality of quails during the first four weeks of life suggesting a long-lasting effect on individual survival in standard rearing conditions. No post-hatching mortality was reported for the I7-16 (12 h/day) treatment in chickens (Collin A., personal communication). Further investigations would be necessary to gain a better understanding of the impact of heat exposure applied during the early hours of embryonic development on the integrity of embryonic development and during the first weeks of life.

TM influenced quail growth, as the weight of hatched quails was significantly increased in TM animals independently from sex. In contrast, other avian TM protocols showed a reduction of body weight in chickens (I7-16, 24 h/day) and in quails (I3, I7 and I13, 2 h/day) at hatching [22,31]. We hypothesize that this effect may have been missed in previous studies. Indeed, in our experiment, we synchronized hatching by incubating C eggs earlier, given that the treated eggs hatched about 12 h sooner than the C eggs. The acceleration of the embryonic development induced by heat has been previously reported in chickens and turkeys [22,42]. In the above mentioned studies where the synchronization was not applied [22,31], the TM animals presumably hatched earlier and therefore spent more time in the hatcher without water or food. Furthermore, TM also impacted quail weight at D25 but not at D35, with TM birds of both sexes being lighter than C quails. The discrepancy between hatching and D25 and the absence of difference at D35 may be explained by compensatory growth, a phenomenon already described in chickens. Indeed, a post-hatching thermal condition procedure applied on 5 day-old chicks induced a body weight reduction in treated animals after the procedure (between day 5 and 8) that was no more visible at D28 [56].

No impact of the TM was observed on internal temperature at D11, D21 and D35, suggesting no dysregulation of the internal temperature homeostasis. However, the TM affected the temperature of non-feathered body parts, with a decrease of the shank surface temperature in TM females compared to C females. In quails, as in other birds, the non-feathered parts of the body such as the shank and the comb contribute to body thermoregulation [24,25] and are involved in heat dissipation during heat exposure [57]. A similar change of surface temperature of a non-feathered peripheral tissue (the comb) has been previously reported for chickens [2,58]. These results suggest that different bird species may share similar mechanisms in response to TM through the modulation of the blood flow system towards peripheral tissues to maintain body temperature homeostasis. The investigation of blood flow, for instance with Doppler measurement [59], may be worth considering to gain new insights about the effect of TM on heat regulation through vascularization.

Previous hematological analyses revealed a TM impact on pCO_2_ and O_2_sat as well as glucose, triglyceride and T3 concentrations at slaughter age (D35) in male chickens [8,10,22]. In quails, we showed a limited impact of TM on quail blood parameters. The TM treatment impacted the blood O_2_ partial pressure and saturation percentage, with an increase observed in TM females when compared with C females, suggesting long-term respiratory adaptation in TM females as previously seen in chickens [10]. Nevertheless, since these parameters were not modified by the HC in interaction with the TM treatment, the implication of this long-term modulation of respiratory physiology in bird response to a heat stress remains unclear [10]. Interestingly, whereas in chickens the impact of TM was preferentially observed in males [10,22], our data suggest that female quails may be more sensitive to the embryonic treatment than males. Thus, the TM may affect the sexes differentially, depending on the species considered.

TM was shown to improve bird tolerance to acute heat exposure in chickens [10,22]. To test the response of TM quails to a heat exposure, we performed a HC at D35 on TM and C quails. While no behavioral signs of distress (panting, reduction of food intake, increase of water intake and prostration) were observed during the HC, several physiological traits were affected by the heat exposure. Notably, HC led to an increase of internal and surface temperatures as well as of the plasma corticosterone concentration, a metabolic hormone associated with stress response in birds [26] and with a decrease of uric acid level in HC birds. These data confirm that the quails exposed to HC displayed markers of heat stress, as previously observed in chickens [10]. Despite no observed panting, we measured a reduction of pO_2_ and O_2_sat with HC, and an increase of TCO_2_ in HC birds suggesting a respiratory insufficiency, as previously reported in chickens [10]. Furthermore, HC impaired the electrolyte balance with lower iCa concentrations and greater K^+^ and HCO_3-_ concentrations in HC birds as compared to RT birds. Various models of heat stress in quails [60] and chickens [10] have been associated with a modulation of electrolyte balance. One of the putative effects of such electrolyte balance disruption is a long-term modification of bone integrity [61] and a modification of egg production for the females [62] that were not assessed in the present study. Moreover, the HC induced a decrease of the total antioxidant status which is in agreement with a previous study in broiler chickens showing that heat stress leads to the induction of oxidative injury and a decreased total antioxidant capacity of the animals [63]. However, we did not find an impact of TM on the total antioxidant capacity as seen in two breeds of chickens by Al-Zghoul et al. [64]. Another effect of HC on quails was an increase of base excess (Beb) and base excess in the extra cellular fluid compartment (Beecf) concentrations. Beb and Beecf were previously shown affected by TM in chickens and may reflect a modification of the acid-base balance due to heat stress [10]. Additionally, while HC tended to be associated with the increase of T4 and glucose concentrations in chickens [10], we observed lower levels of these two parameters in HC quails, suggesting that different hormonal and metabolic mechanisms mediate heat stress response in quails. These responses may be species-specific and/or could be due to differences in the experimental designs of TM and HC between studies. Indeed, in addition to comparing different TM and HC procedures, the major difference relative to the specific responses of each species may be linked to the genetic selection of each agronomic line. Despite the fact that Japanese quails were selected for meat and egg production, the selection was less extensive than for chickens, leading to the observation that quails are physiologically more resistant than chickens to diseases and environmental changes [65].

In chickens, a heat acclimation marker reported in TM animals was a lower increase of plasma corticosterone concentrations at the end of the HC [10]. In our experimental model, no such regulation was observed, since the plasma corticosterone concentration was impacted by the HC similarly in C and TM experimental groups. Nevertheless, out of all the physiological parameters analyzed, two appeared to be impacted by the HC in interaction with the TM and the sex: the beak surface temperature and the concentration of Na^+^. Beak surface temperature increase induced by the HC was less important in TM females, suggesting a differential regulation of heat exchanges to maintain the internal temperature homeostasis in the case of heat stress in TM birds. Similar sex-specific mechanisms of thermoregulation in case of heat stress were also reported in humans [66]. The Na^+^ blood concentration was lowest in HC-TM females and it was highest in RT-C males. This result suggests that there may be a sex-dependent modification of electrolyte balance in response to heat exposure in quail. Despite the fact that no impact on plasma Na^+^ concentration was observed on heat-stressed TM chickens [10], one study showed a reduction of Na^+^ in a case of acute increase of environmental temperature in male broiler chickens [67]. In human, Baker and colleagues have revealed a difference of Na^+^ sweat concentrations between males and females during moderate exercise-heat stress, suggesting a differential regulation of the blood electrolyte balance to maintain thermal homeostasis [68]. Although no sweating process is observed in birds, such similar difference in the regulation of electrolyte balance between sexes could be involved in the body thermal homeostasis. Together, despite the absence of clear evidence of adaptation to heat stress in TM quails, unlike previous reports for chickens [10,22], our data shows that TM impacted several physiological parameters of quails in response to heat, especially in females, that may contribute towards an adapted response of TM quails to heat stress.

## Conclusions

Thermal manipulation during embryogenesis had a long-lasting impacts on Japanese quail development and physiology, including an effect on weight and mortality. Surface temperature of the shank was also impaired, suggesting a modulation in the capacity to regulate heat exchanges with the environment. Whereas hematological analyses revealed an impact of TM on blood oxygen parameters preferentially in females, our data also showed a differential response of TM birds when challenged by moderate heat exposure in this same sex. A reduction in the period of the TM treatment which does not encompass the early stages of the embryogenesis may be worth investigating to mitigate detrimental effects including mortalities observed TM in quails. In addition, a more acute heat challenge or differently timed may be worth considering to reveal the acclimation potential of TM. Nevertheless, this procedure remains a valuable experimental model to explore the physiological and molecular mechanisms involved in the long-term impact of environmental changes during the embryogenesis in vertebrates.

## Supporting information

S1 Fig **Evaluation of surface temperatures (°C) from infrared pictures at 35 days of age (A-B).** (A) The maximal temperature of the head corresponding to the ear temperature and the surface temperature of the eye were evaluated respectively as the hottest and the coolest points on the head. Beak surface temperature was evaluated as the temperature mean of the beak area. (B) Leg surface temperature was evaluated as the mean of a line drawn along the entire shank of the bird.
(TIF)

S1 Table Individual data of egg weight and embryo development until hatching. Treat_emb: incubation treatment applied to the egg. C: control. TM: thermal manipulation during embryonic incubation. Parents: identification number of the quail couple associated with the considered egg.
(XLSX)

S2 Table Individual data of survival between hatching and day 28. Treat_emb: incubation treatment applied to the egg. C: control. TM: thermal manipulation during embryonic incubation. Parents: identification number of the quail couple associated with the considered egg. 0: alive; 1: dead at D28.
(XLSX)

S3 Table Individual data of weight, temperature, blood parameters and hormone and metabolite concentrations. Parents: identification number of the quail couple associated with the considered individual. Treat_emb: incubation treatment applied to the egg. C: control. TM: thermal manipulation during embryonic incubation. Treat_D35: heat challenge treatment applied to the individual at D35. RT: room temperature. HC: heat challenge. Sex: sex of the animal (1: male, 2: female).
(XLSX)

S1 File Internal temperature (°C) analysis at D11 and D21. (DOCX)
