# Supplementary figures and images for: Embryonic thermal manipulation has short and long-term effects on the development and the physiology of the Japanese quail
Source: PLoS One. 2020 Jan 23;15(1):e0227700. doi: 10.1371/journal.pone.0227700 (PMC6977749; doi:10.1371/journal.pone.0227700)

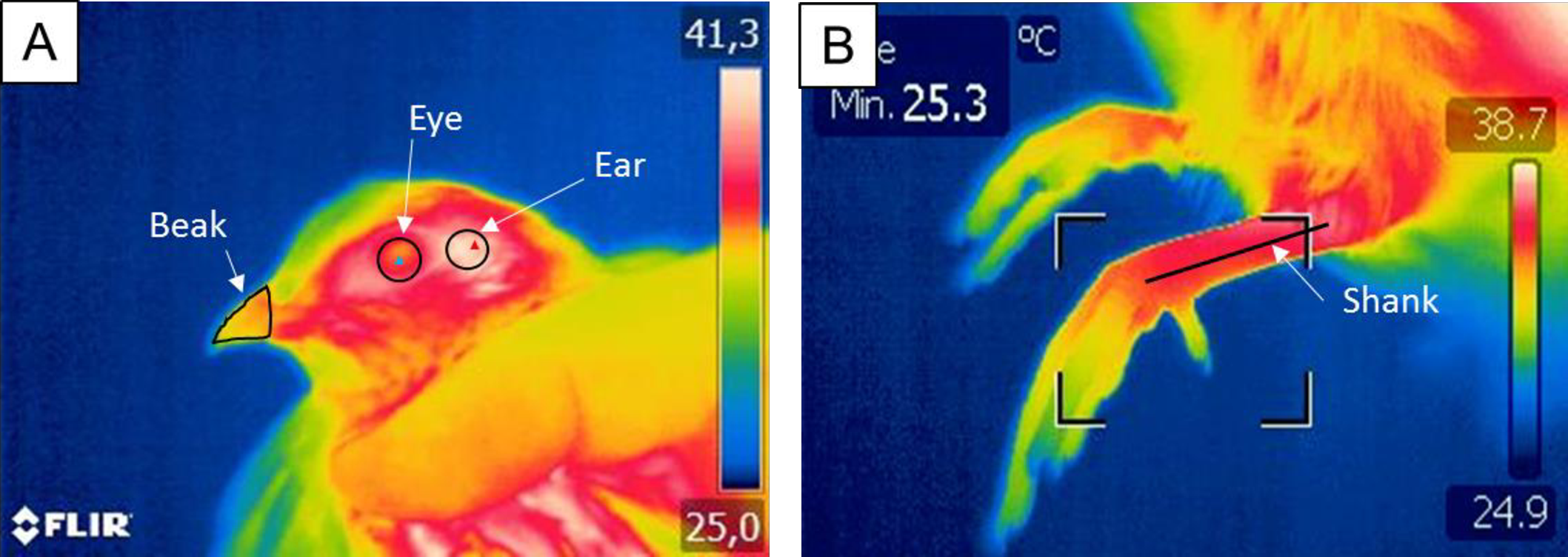

Supplement: S1 Fig — Evaluation of surface temperatures (°C) from infrared pictures at 35 days of age (A-B). (A) The maximal temperature of the head corresponding to the ear temperature and the surface temperature of the eye were evaluated respectively as the hottest and the coolest points on the head. Beak surface temperature was evaluated as the temperature mean of the beak area. (B) Leg surface temperature was evaluated as the mean of a line drawn along the entire shank of the bird. (TIF) [file pone.0227700.s001.tif]
