# Supplementary material for: Embryonic thermal manipulation has short and long-term effects on the development and the physiology of the Japanese quail
Source: PLoS One. 2020 Jan 23;15(1):e0227700. doi: 10.1371/journal.pone.0227700 (PMC6977749; doi:10.1371/journal.pone.0227700)
Supplement: S1 File — (DOCX) [file pone.0227700.s005.docx]

### Internal temperature analysis at D11 and D21

Only the animals hatched at I17 were considered for the temperature analysis at D11 and D21. Consequently, the statistical model was developed to analyze the impact of the sex and the TM on internal temperature at D11 and D21 independently with 26 to 42 animals per sex and per condition.

The following linear model (model 1) was used:

Y_ijkl_ = α_i_ + β_j_ + (αβ)_ij_ + ε_ij_

where Y_ijkl_ is the observed variable measured on the animal l, α_i_ is the fixed effect of sex (i = female and male), β_j_ is the fixed effect of embryonic treatment TM (j = C, TM), (αβ)_ij_ is the interaction effect of sex and TM, ε_ij_ is the random residual term ~ N(0, σ^2^).

**Table:** **Effects of sex and thermal manipulation on internal temperature at D11 and D21.** Individual data are presented in Table S3.

|  | **Statistics (p-value)** | | |
| --- | --- | --- | --- |
|  | **Sex** | **TM** | **Sex*TM** |
| D11 | 0.390 | 0.956 | 0.471 |
| D21 | 0.930 | 0.141 | 0.198 |
